# Evolutionary analyses reveal independent origins of gene repertoires and structural motifs associated to fast inactivation in calcium-selective TRPV channels

**DOI:** 10.1038/s41598-020-65679-6

**Published:** 2020-05-26

**Authors:** Lisandra Flores-Aldama, Michael W. Vandewege, Kattina Zavala, Charlotte K. Colenso, Wendy Gonzalez, Sebastian E. Brauchi, Juan C. Opazo

**Affiliations:** 1 Instituto de Fisiología, Facultad de Medicina, Universidad Austral de Chile, Valdivia, Chile; 2 Programa de Doctorado en Ciencias mención Biología Celular y Molecular, Escuela de Graduados, Facultad de Ciencias, Universidad Austral de Chile, Valdivia, Chile; 3 Millennium Nucleus of Ion Channels-Associated Diseases (MiNICAD), Valdivia, Chile; 4 Department of Biology, Eastern New Mexico University, Portales New Mexico, USA; 5 Instituto de Ciencias Ambientales y Evolutivas, Facultad de Ciencias, Universidad Austral de Chile, Valdivia, Chile; 6 Center for Bioinformatics and Molecular Simulations (CBSM) University of Talca, Talca, Chile

**Keywords:** Evolutionary genetics, Molecular evolution

## Abstract

Essential for calcium homeostasis, TRPV5 and TRPV6 are calcium-selective channels belonging to the transient receptor potential (TRP) gene family. In this study, we investigated the evolutionary history of these channels to add an evolutionary context to the already available physiological information. Phylogenetic analyses revealed that paralogs found in mammals, sauropsids, amphibians, and chondrichthyes, are the product of independent duplication events in the ancestor of each group. Within amniotes, we identified a traceable signature of three amino acids located at the amino-terminal intracellular region. The signature correlates with both the duplication events and the phenotype of fast inactivation observed in mammalian TRPV6 channels. Electrophysiological recordings and mutagenesis revealed that the signature sequence modulates the phenotype of fast inactivation in all clades of vertebrates but reptiles. A transcriptome analysis showed a change in tissue expression from gills, in marine vertebrates, to kidneys in terrestrial vertebrates. Our results highlight a cytoplasmatic structural triad composed by the Helix-Loop-Helix domain, the S2-S3 linker, and the TRP domain helix that is important on modulating the activity of calcium-selective TRPV channels.

## Introduction

In vertebrates, epithelial calcium absorption is a crucial physiological process needed to maintain Ca^2+^ homeostasis. Epithelia consist of a continuous layer of individual cells where calcium absorption is maintained by two routes, the transcellular and paracellular pathways^1,2^. Tight junctions regulate the passive passage of ions and molecules through the paracellular pathway^2^. Calcium permeation through this pathway constitutes a major route for Ca^2+^ absorption under normal physiological conditions^1^. On the other hand, the transcellular pathway implicates controlled Ca^2+^ movement through epithelial barriers and occurs against a concentration gradient in normal conditions. It is an active and saturable process, maintained by an array of transporters, pumps, and ion channels^1^.

TRPV5 and TRPV6 are calcium-selective ion channel members of the Transient Receptor Potential (TRP) gene family^3^. These proteins, expressed at the apical membrane of Ca^2+^ transporting epithelia, serve as entry channels in transepithelial Ca^2+^ transport^3^. In agreement with their physiological role, TRPV6-EphB6 double knockout mice have impaired Ca^2+^ homeostasis, evidenced by poor weight gain, decreased bone mineral density, and reduced fertility^4^. However, more recent research using TRPV6 null mice suggests that the channel becomes relevant only when dietary calcium supply is low^5^. On the other hand, TRPV5 null mice present less severe physiological consequences, nonetheless, the absence of TRPV5 channels causes hypercalciuria, compensatory hyperabsorption of dietary calcium, and abnormal bone thickness^6^. At negative membrane potentials, calcium-selective TRPV channels facilitate the passage of calcium ions to the cytoplasm. Calcium selectivity in TRPV5 and TRPV6 is accompanied by different mechanisms of Ca^2+^-dependent inactivation, helping to modulate Ca^2+^ entry during the first steps of transcellular transport^3,7^. One major difference between mammalian TRPV5 and TRPV6 channels rests in their distribution among tissues. Mammalian TRPV5 is predominantly expressed in the distal convoluted tubule and connecting tubule of the kidney, where it is likely to play a role in Ca^2+^ reabsorption. In contrast, mammalian TRPV6 is expressed in a variety of tissues; it is found not only in the Ca^2+^ absorbing epithelia of the gut but in placenta, pancreas, and prostate, suggesting that the physiological roles of TRPV6 might not be limited to the maintenance of Ca^2+^ homeostasis^8^.

Among vertebrates, it has been suggested that mammalian TRPV5 and TRPV6 channels originated from a duplication event in the last common ancestor of the group, between 312 and 177 million years ago^9,10^. This would suggest that these genes belong to the mammalian clade exclusively and are not orthologs to TRPV5 and TRPV6 channels of other vertebrate groups. Besides these observations, not much else is known about the evolutionary history of these channels in vertebrates. Here we performed a comparative study of calcium-selective TRPV channels on representative species of all main groups of vertebrates. Thorough phylogenetic analyses in combination with functional assays allowed us to explore their evolutionary history and the evolution of functional features.

## Results

### Phylogenetic, primary sequence, and synteny analyses support the monophyly of the calcium-selective TRPV channels in vertebrates

**Figure 1:**
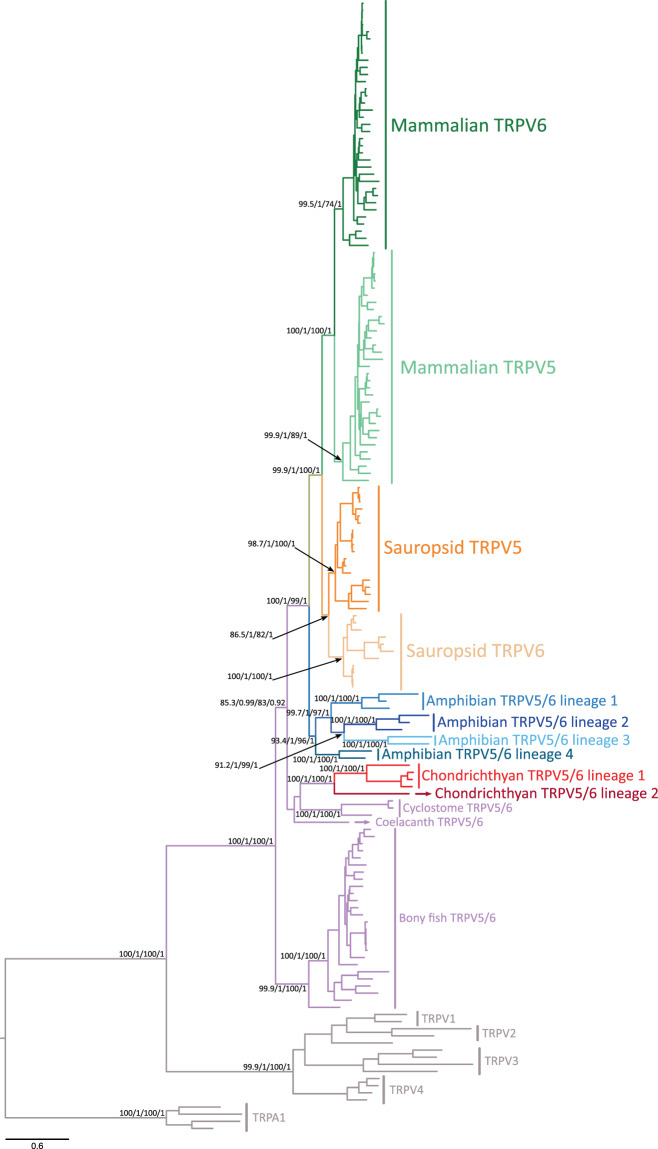
Maximum likelihood tree depicting evolutionary relationships among calcium-selective TRPV channels of vertebrates. Numbers above the nodes correspond to support from the Shimodaira-Hasegawa approximate likelihood-ratio test, aBayes, maximum likelihood ultrafast bootstrap and posterior probability values. TRPV1, TRPV2, TRPV3, TRPV4 and TRPA1 were used as outgroups. The scale denotes substitutions per site and colors represent gene lineages. The alignment used to build this phylogenetic tree, as well as, a phylogeny with species names for each terminal branch is found in the supplementary material section.

To understand the duplicative history of calcium-selective TRPV channels, we first reconstructed a phylogenetic tree including sequences from species of all major groups of vertebrates. According to our maximum likelihood and Bayesian analyses, all node support measurements reached maximal values (100/1/100/1) for the monophyly of the group that includes all vertebrate calcium-selective TRPV channels (Fig. 1). This indicates that all sequences included in our phylogenetic reconstruction derived from an ancestral sequence that was present in the genome of the vertebrate ancestor between 676 and 615 million years ago. A closer inspection of the multiple sequence alignment (MSA) supports the monophyly of the group as several conserved residues can be easily mapped (Supplementary Figure 1). Gaps and/or deletions in the alignment -introduced by the outgroups (TRPV1–4)- are not shared by any of the ingroup sequences (Fig. 1; Supplementary Figure 1). The differences between the TRPV1–4 and TRPV5–6 clades can be traced to specific regions, including the ankyrin repeat domain (ARD), the Helix-loop-Helix domain (HLH), the intracellular linker between transmembrane segments 2 and 3 (S2–S3), and the pore domain (PD) (Supplementary Figure 1). Moreover, synteny analyses also provide support for monophyly of the calcium-selective TRPV clade. Genes encoding TRPV5 and TRPV6 channels are located in a conserved genomic region, flanked by EPHB6 and KEL, suggesting that this genomic location was present early in vertebrate evolution and has remained relatively well conserved (Fig. 1; Supplementary Figure 2). Thus, phylogenetic, primary sequence, and synteny analyses provide strong support for the monophyly of calcium-selective TRPV channels in vertebrates.

### Phylogenetic evidence for the independent origin of calcium-selective TRPV channels in mammals, sauropsids, amphibians, and chondrichthyes

**Figure 2:**
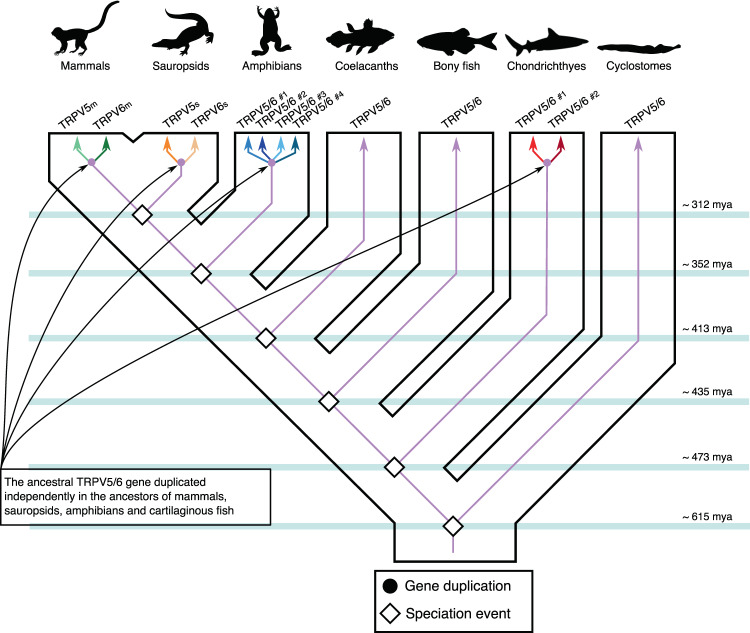
An evolutionary hypothesis regarding the evolution of the calcium-selective TRPV channels in vertebrates. According to our model the last common ancestor of vertebrates had a repertoire of one calcium-selective TRPV gene (TRPV5/6), a condition that has been maintained in cyclostomes, bony fish and coelacanths. In the ancestors of chondrichthyes, amphibians, sauropsids and mammals the single gene copy underwent independent duplication events giving rise to a more diverse repertoire of calcium-selective TRPV channels. In the case of chondrichthyes, sauropsids and mammals the single gene copy present in the ancestor of each group underwent a single duplication event-giving rise to a repertoire of two calcium-selective TRPV genes. In the case of amphibians, multiple duplication events in the ancestor of the group gave rise to a repertoire of four calcium-selective TRPV genes, for more details see Fig. 1. Divergence times were obtained from www.timetree.org. Vertebrate silhouette images were obtained from PhyloPic (http://phylopic.org/).

Within the calcium-selective TRPV clade, our phylogenetic analyses suggest not a single but four independent expansion events during the evolutionary history of vertebrates (Figs. 1 & 2). These can be noticed in our phylogenetic tree as duplicated copies in mammals, sauropsids, amphibians, and chondrichthyes are recovered sister to each other within each clade. For example, the sister group relationship between the mammalian TRPV5 and TRPV6 gene lineages is maximally supported (100/1/100/1) (Fig. 1), reinforcing the hypothesis that the duplication event that gave rise to these copies occurred in the mammalian ancestor (Fig. 2), as previously suggested^9,10^. Similarly, the sister group relationship between the sauropsid gene lineages is also well supported (86.5/1/82/1) (Fig. 1), indicating that a duplication event occurred in the sauropsid ancestor (Fig. 2). Amphibians possess the most diverse repertoire of TRPV5/6 paralogs of all vertebrates where four well-supported gene lineages were recovered (Figs. 1 & 2). According to our phylogenetic tree, these amphibian gene lineages likely originate in the ancestor of the group (Fig. 2). Such ancestor can be traced back to the carboniferous period, where amphibians were the dominant vertebrate terrestrial species^11^. Similar to the situation described for mammals, sauropsids, and amphibians, our phylogenetic reconstruction supports a scenario in which the chondrichthyan (e.g. sharks, rays, and chimaeras) gene repertoire of calcium-selective TRPV channels was originated via gene duplication in the ancestor of the group (Figs. 1 & 2). In contrast, the condition of a single gene copy (referred here as TRPV5/6) is present in cyclostomes, bony fish, and coelacanths (Fig. 1). Thus, our results indicate that the gene repertoire observed in mammals, sauropsids, amphibians, and chondrichthyes originated independently in the ancestors of each group (Fig. 2), suggesting that TRPV5 and TRPV6 in these groups are not 1:1 orthologs (Fig. 2).

### Birds retained only one calcium-selective TRPV channel

**Figure 3:**
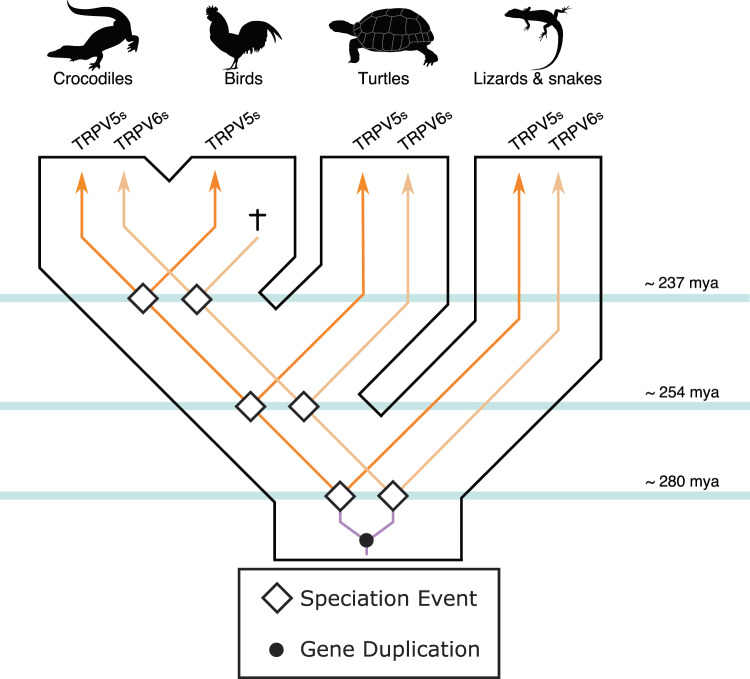
An evolutionary hypothesis regarding the evolution of the calcium-selective TRPV channels in sauropsids. According to this model the last common ancestor of sauropsids had a repertoire of one calcium-selective TRPV gene (TRPV5/6), which underwent a duplication event giving rise to a repertoire of two genes. During the radiation of the group the repertoire of two calcium-selective TRPV channels was maintained in crocodiles, turtles, lizards and snakes. However, in the ancestor of birds one of the genes was lost (sauropsid TRPV6), and the condition of a single gene copy was inherited by all descendant species. Vertebrate silhouette images were obtained from PhyloPic (http://phylopic.org/).

When compared with other vertebrates, the case of sauropsids represents a special case of study. As in other tetrapods, the single gene copy present in the ancestor of the group underwent a duplication event originating a repertoire of two calcium-selective TRPV genes (Figs. 1 & 3). However, during the radiation of the group, sauropsid TRPV5 was retained in all major lineages whereas sauropsid TRPV6 was found absent in birds (Fig. 3). A comparison of the genomic regions in chicken and crocodile showed remaining exons of the sauropsid TRPV6 gene in the genome of chicken (Supplementary Figure 3). This suggests first that the gene present in birds corresponds to a sauropsid TRPV5 and not to a sauropsid TRPV6 as it has been previously suggested in the literature^9,10^. The fact that birds can maintain calcium homeostasis according to their physiological requirements with just one of the calcium-selective TRPV channel could mean that a repertoire of two genes represent a case of functional redundancy in birds. In such case, the loss of one of the calcium-selective TRPV channels in the ancestor of birds could be understood as part of a stochastic process where the loss of the sauropsid TRPV6 is compensated by the retention of the sauropsid TRPV5 channel. This idea is supported by the fact that there are other vertebrate groups that maintain normal calcium homeostasis with just one calcium-selective TRPV channel^12^. However, we can’t rule out a purifying selection of the channel set that might have been driven by environmental or dietary stress.

### A three amino acid motif in the HLH domain is important in fast inactivation

**Figure 4:**
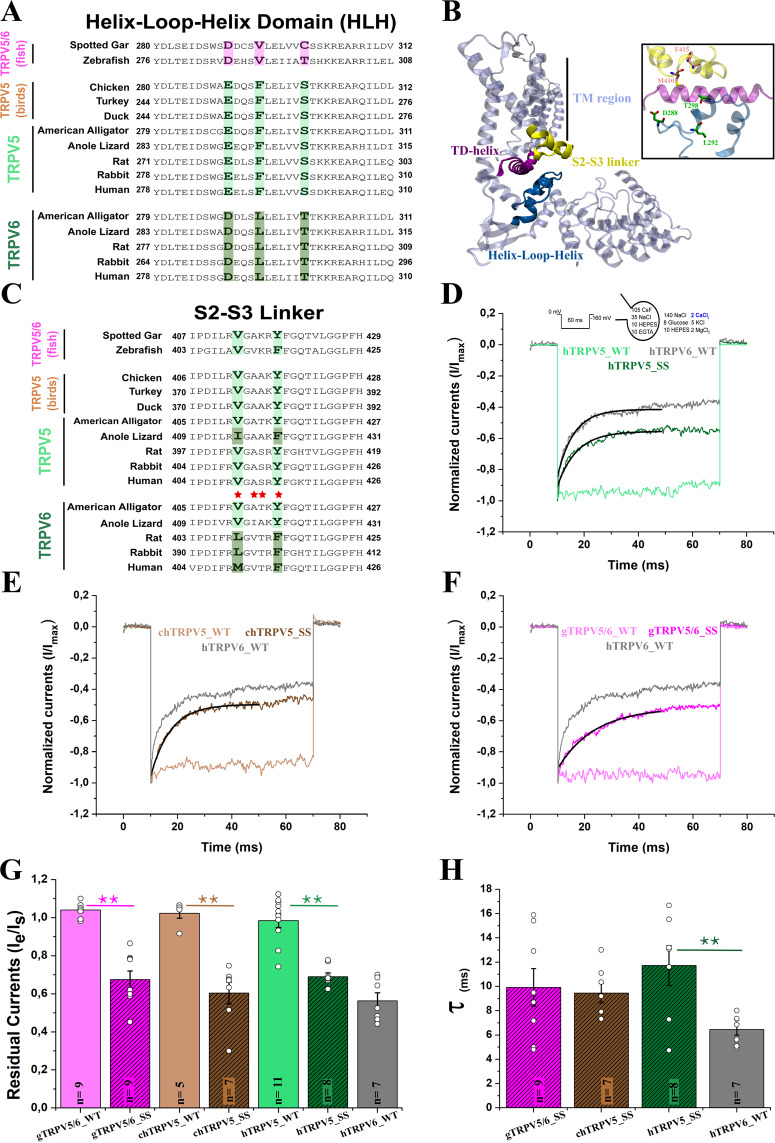
Specific amino acid replacements in the HLH domain and S2-S3 linker correlate with the fast inactivation phenotype. (**A**) Amino acid sequence alignment of the HLH domain. Conserved residues in TRPV5/6 (pink), TRPV5-type (light green) and TRPV6-type (dark green) channels are highlighted. (**B**) Structure of one subunit of the human TRPV6 channel (hTRPV6; PDB: 6E2F)^22^ showing the structural triad HLH domain (blue), S2-S3 linker (yellow) and TD-helix (purple). TM region: Transmembrane region. The inset shows the location of the signature side chains in TRPV5. (**C**) Amino acid sequence alignment of the S2-S3 linker of the TRPV5-type, TRPV6-type and TRPV5/6 channels. Non-inactivating channels show conserved residues (light green) that differ from residues present in mammalian TRPV6 channels (dark green). Red stars indicate residues located at the S2-S3 linker previously mutated by Nilius *et al*.^17^, affecting fast inactivation in a mammalian TRPV6 channel. (**D**) Normalized whole cell current traces recorded from transiently transfected HEK-293T cells expressing hTRPV5, hTRPV6, and engineered TRPV5 (hTRPV5_SS; E288D; F292L; S298T) containing the three-residue signature sequence at the HLH domain of TRPV6 channels. (**E**) Normalized whole cell current traces recorded from the chicken TRPV5 (chTRPV5_WT) and its triple mutant (chTRPV5_SS; E290D; F294L; S300T) containing the three-residue signature sequence at the HLH domain of TRPV6 channels and human TRPV6 channel (hTRPV6 WT). (**F**) Normalized whole cell current traces recorded from the spotted gar TRPV5/6 (gTRPV5/6_WT) and its mutant (chTRPV5/6_SS; V294L; C300T) containing the three-residue signature sequence at the HLH domain of TRPV6 channels and human TRPV6 channel (hTRPV6 WT). (**G**) Pooled data comparing the residual currents (see methods). (**H**) Pooled data comparing the inactivation time constants for inactivating clones (see methods). All current traces were recorded in the presence of 2 mM extracellular Ca^2+^ and in response to a voltage pulse at −160 mV for 60 ms from Vh=0 mV (Panels D; E; F). Black line indicates the fitting to a single exponential function (**D,E,F**). All bars represent mean value, errors represent S.E. and white dots represent values for each experiment (Panels G; H). ** represents p = 0.01 and *p = 0.05.

A closer inspection of the alignment used in our phylogenetic reconstruction revealed a remarkably strong signature sequence that correlates well with gene duplication events in amniotes. This signature is composed of three amino acids located at the N-terminal helix-loop-helix (HLH) domain in mammals and sauropsids (Fig. 4A). According to recently published structures^13,14^, the HLH domain sits close to the S2-S3 intracellular linker and conveniently adjacent to the TRP domain helix (TD-helix), a known modulator of TRP channel gating^15,16^ (Fig. 4B). Our sequence alignment also revealed a conserved pair of amino acids located in the S2-S3 linker. However, in contrast to the signature found at the HLH domain, the amino acids in the linker correlate with the duplication events only in mammals (Fig. 4C). Previous studies have identified both residues at the S2-S3 linker as structural modulators of fast-inactivation in mammalian TRPV6 channels^17^. Considering an independent origin for these channels the presence of these signatures -distant in the primary sequence but in close contact in the folded structure- suggests a functional association between the S2-S3 linker, the HLH domain, and the TD-helix. A multiple sequence alignment performed to the elements of the triad contrasts the variability observed at the HLH domain and S2-S3 linker with the conservation of the TD-helix (Supplementary Figure 4). The TD-helix is well conserved within the TRPV family and the TRP family in general^18^. In contrast, the S2-S3 linker and the HLH domain display a pattern of residue conservation that is lower in bony fish/amphibians and higher in sauropsids/mammals somewhat suggesting co-evolution between the two regions (Supplementary Figure 4).

From a functional perspective, mammalian TRPV6 channels exhibit a characteristic two-component calcium-dependent inactivation^7^. In calcium-selective TRPVs, slow inactivation is determined by the binding of the Ca^2+^-Calmodulin complex to the C-terminal region of the channel and likely operates by the interaction of calmodulin’s C-lobe with the intracellular pore of the channel^19–22^. Slow inactivation is present in both TRPV5 and TRPV6 channels from mammals. In contrast, fast inactivation depends solely on the presence of calcium ions and has been reported only in mammalian TRPV6 channels^7,17^. Because of the role of the S2-S3 linker and the TD-helix in fast inactivation^17,23^, we question whether these three amino acids at the HLH domain, represent not only a signature associated to duplication events but are also linked to a functional trait. To this end, we transferred the three amino acid signature found in the HLH domain of the human TRPV6 channel (hTRPV6) into the human TRPV5 channel (hTRPV5). Whole cell electrophysiological recordings showed that engineered hTRPV5 channels (hTRPV5_SS: E288D, F292L, S298T) now feature a robust fast inactivation in the presence of 2 mM extracellular [Ca^2+^] (Fig. 4D,G). Wild type avian channels (*Gallus gallus*; chTRPV5) showed the absence of fast inactivation, as seen in mammalian TRPV5 channels (Fig. 4E). In agreement with our previous results, engineered chTRPV5 channels (chTRPV5_SS: E290D, F294L, S300T) containing the amino acid signature from hTRPV6 now feature robust fast inactivation, mimicking mammalian TRPV6 channels (Fig. 4E,G). Thus, our functional evidence shows that birds only kept a non-fast inactivating channel in their genomes. We also investigated inactivation in channels from vertebrates that possess the condition of single gene copy (e.g., spotted gar, *Lepisosteus oculatus*; gTRPV5/6). In the presence of 2 mM extracellular [Ca^2+^] we observed that fast-inactivation is absent in gTRPV5/6 (Fig. 4F). Moreover, as seen in mammals and birds, fast inactivation can be easily introduced by mutagenesis at the HLH domain (gTRPV5/6_SS: V294L, C300T) (Fig. 4F,G). An inspection of the kinetics of the inactivation process helps to understand gating mechanisms associated to inactivation. Although all HLH mutants showed a similar time constant for fast inactivation (10–12 ms; Fig. 4H), this nearly doubles the kinetics observed in wild type hTRPV6 (6 ms). This suggests an additional element modulating inactivation, common to all but likely better tuned in hTRPV6, that is still not yet identified. Overall, our data highlights the importance of the HLH domain and suggest a strong functional connection between the HLH and fast-inactivation.

### Calcium-selective TRPVs from reptiles

**Figure 5:**
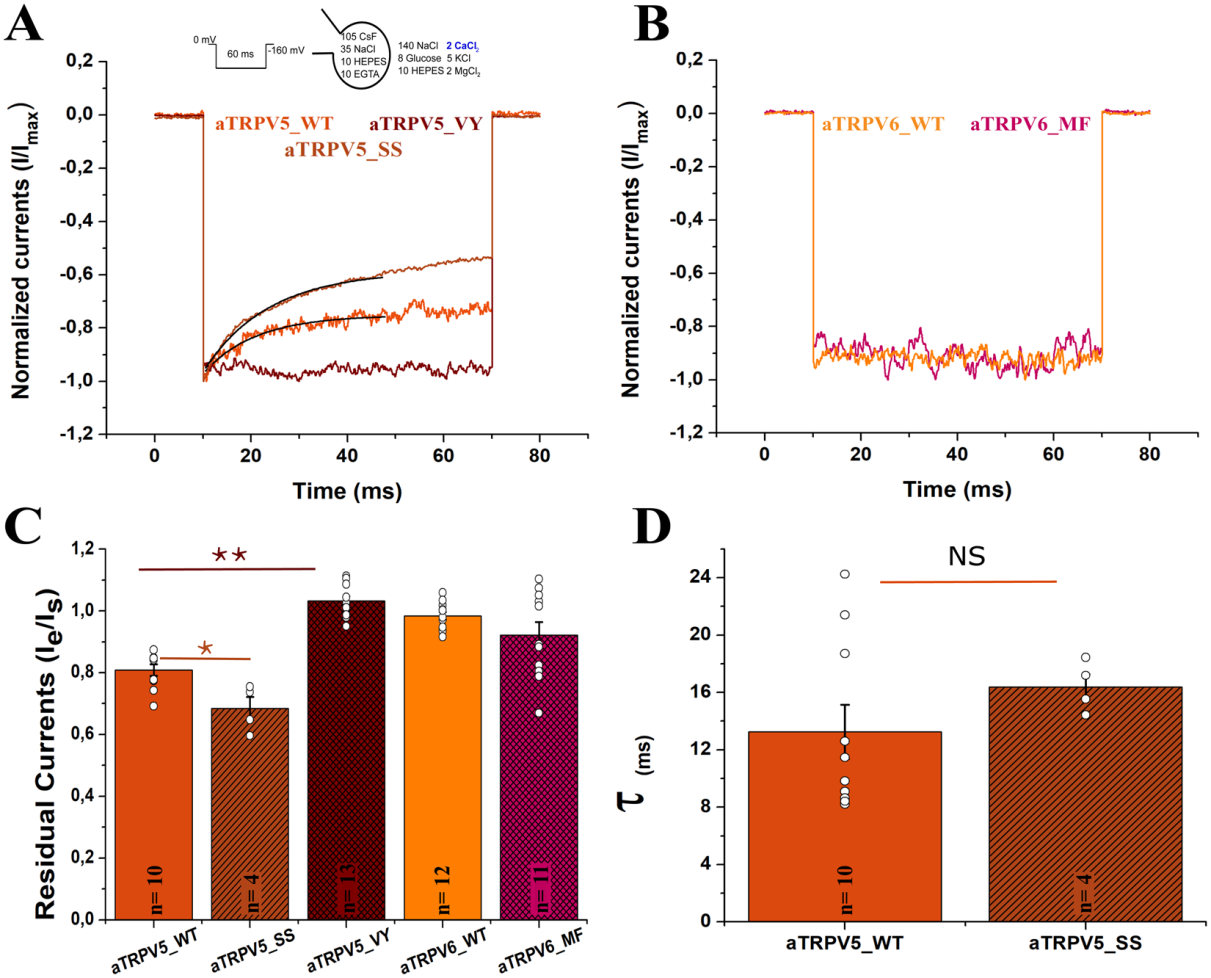
Calcium-selective TRPV6 from reptiles lack a robust inactivation phenotype. (**A**) Normalized whole cell current traces recorded from transiently transfected HEK-293T cells expressing anole lizard TRPV5 channel wild type (aTRPV5 WT), containing the three-residue signature sequence of TRPV6 channels (aTRPV5_SS; E293D; F297L; S303T), and containing the amino acid signature corresponding to TRPV5 channels at the S2-S3 linker (aTRPV5_VY; I415V; F420Y). (**B**) Normalized whole cell current traces recorded from the anole lizard TRPV6 channel wild type (aTRPV6 WT) and containing the amino acid signature corresponding to human TRPV6 (aTRPV6_MF) (**C**) Pooled data comparing the residual currents. (**D**) Pooled data comparing the inactivation time constants for inactivating clones. All current traces were recorded in the presence of 2 mM extracellular Ca^2+^ and in response to a voltage pulse at −160 mV for 60 ms from Vh=0 mV (Panels A,B). Black line indicates the fitting to a single exponential function (**A**). All bars represent mean value, errors represent S.E. and white dots represent residual current value for each experiment (Panels C,D). ** represents p = 0.01 and *p = 0.05.

Assuming a strict correlation between the duplication signature at the HLH domain and the phenotype of fast inactivation, anole lizard’s TRPV5 channel (aTRPV5) should not fast-inactivate according to their sequence profile (Fig. 4A). In contrast, the residues at the S2-S3 linker (I415, F420) somewhat correspond to fast-inactivating mammalian TRPV6 channels (M410, F415) (Fig. 4C). In all the inactivating channels tested, including mutants in birds and bony fish, fast inactivation causes about 40% loss in the residual current within 50 ms. Electrophysiological recordings showed that the anole lizard’s TRPV5 channel elicits a modest fast inactivation where only 20% of the residual current is lost at steady state (Fig. 5A). By introducing the sequence of non-inactivating channels at the S2-S3 linker (aTRPV5_VY: I415V, F420Y) we managed to completely abolish fast inactivation (Fig. 5A). The sole introduction of HLH signature in the anole lizard’s TRPV5 channel (aTRPV5_SS: E293D, F297L, S303T) produced a robust inactivation phenotype, similar to the one observed in the mammalian TRPV6 channel (Fig. 5A,C). Moreover, we found that the time constant of inactivation in both aTRPV5 and aTRPV5_SS (Fig. 5D) was similar to other TRPV5-type mutants. All together, these results support our hypothesis of a functional association between the HLH domain and the S2-S3 linker.

However, in contrast with the flexibility to adopt a new inactivation phenotype observed in all TRPV5-type channels analyzed (including aTRPV5), the anole lizard TRPV6 channel (aTRPV6) showed to be an exception that is unsusceptible to inactivation. In this case, even the presence of both signatures shared by inactivating channels (aTRPV6_MF: HLH: D293, L297, T303; linker S2-S3: V415M, Y420F) was not enough to alter the non-inactivating nature of the channel (Fig. 5B,C). It has been reported that at high extracellular calcium (10 mM) both human channels, hTRPV5 and hTRPV6, feature fast inactivation^7^. In our hands this holds true for calcium-selective TRPV channels from human, but not observed in sauropsids (Supplementary Figure 5). These results suggest the presence of an additional modification in sauropsids, preventing inactivation by either modifying calcium binding or coupling. Such additional modification shouldn’t be present in the single copy gTRPV5/6, where high extracellular calcium readily induces fast inactivation (Supplementary Figure 5). Thus, we suggest that sauropsids in general require a non-fast inactivating channel set. In one hand reptiles introduced structural modifications making these channels insensitive to calcium variations and/or becoming resistant to inactivation after calcium binding, while birds just retained a non-inactivating channel.

### Change of the expression profile from gills to kidneys in vertebrates

**Figure 6:**
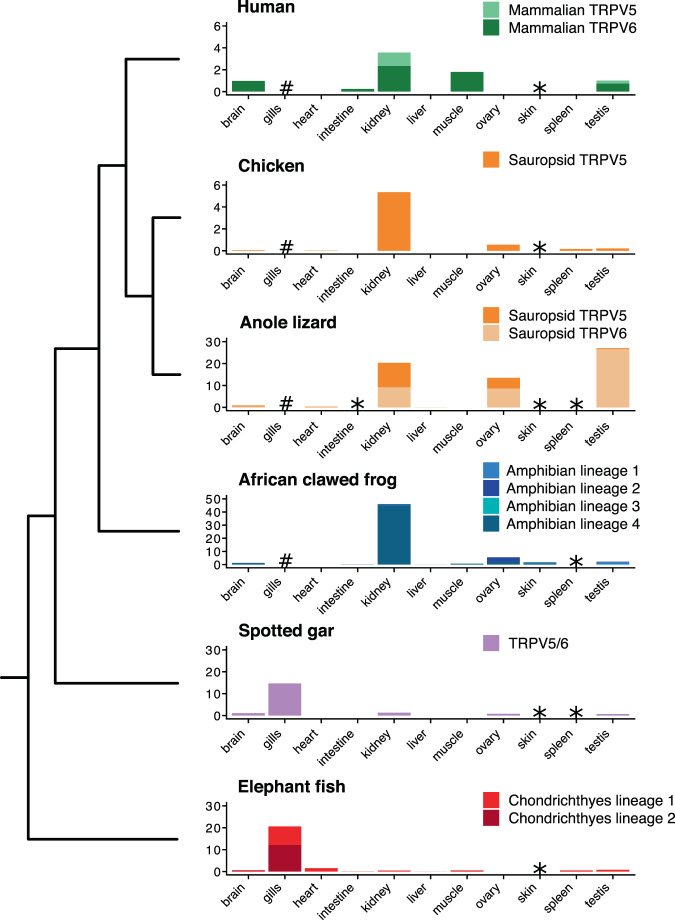
Comparative expression profiles of calcium-selective TRPV channels of vertebrate across multiple tissues. mRNA levels were estimated from public RNA-Seq libraries. Asterisks indicate data were not available for that species and tissue, and hashes indicate the tissue was absent. Gene expression was measured in transcript per million (TPM).

Based on expression profiles and inactivation properties it has been suggested that calcium-selective TRPV channels in mammals use a strategy in which the territories of expression are divided. While the mammalian TRPV6 channel should play a major role in Ca^2+^ absorption, mammalian TRPV5 would be mainly dedicated to calcium reabsorption in the kidney^3,8^. This correlates well with their expression profiles; while mammalian TRPV6 channels are expressed in a variety of tissues (including the kidney, intestine, placenta, pancreas, and prostate), mammalian TRPV5 is restricted to the distal convoluted tubule and connecting tubule in the kidney^3,8^. In contrast to mammals, much less is known regarding the expression of these genes in other vertebrates. We then characterized the transcription profiles of calcium-selective TRPV channels in representative species of vertebrates (Fig. 6).

In agreement to what has been reported in the literature, our results show a localized expression for human TRPV5 channels while human TRPV6 is expressed in a variety of tissues (Fig. 6). Interestingly, both genes are well expressed in gonads, something that was found common to all surveyed species (Fig. 6). In support of previous reports^12^, we observed that aquatic vertebrates (i.e. spotted gar and elephant fish) mostly express calcium-selective TRPV channels in their gills, no matter the number of gene copies present in their genomes (Fig. 6). The expression pattern observed in elephant fish suggests that the expansion of the gene repertoire did not give rise to a pattern in which the territories of expression were divided but rather served to increase the number of channels expressed in the gills (Fig. 6). In contrast, the preferential expression of a single calcium-selective TRPV in kidney starts to be obvious in amphibians, where one of the four gene lineages present in their genome is clearly more abundant in this organ (Fig. 6). The channels representing the other three gene lineages are present in other tissues such as skin, brain, and testis (Fig. 6). This expansion in both gene repertoire and territories of expression might suggest early adaptations needed for terrestrial lifestyle^24^. Thus, it seems that together with the conquest of land, one of the copies is mainly expressed in kidneys while others are expressed in a variety of tissues.

## Discussion

Our study shows that the evolutionary history of calcium-selective TRPV channels of vertebrates is mainly characterized by events of gene birth and death. According to our results, essential genes for calcium homeostasis have been duplicated four independent times during the evolutionary history of vertebrates. Our analysis suggests that mammals, sauropsids, amphibians, and chondrichthyes originated their repertoire of calcium-selective TRPV genes independently in the ancestor of each group, indicating that TRPV5 and TRPV6 in these groups are not 1:1 orthologs. This observation implies that calcium-selective TRPV channels from these groups have different evolutionary origins in different vertebrate groups. The independent origin of genes seems not to be uncommon in nature. Examples of these are ß-globin genes in vertebrates^25–27^, miRNA genes in monocots and brassicales^28^, MHC class II genes in primates^29^, growth hormone genes in anthropoid primates^30,31^, growth differentiation factors (GDF1 and GDF3) in mammals and anurans^32^, among others. In these cases the function of gene products serves to the same purpose, however, gene repertoires are not directly comparable as, strictly speaking, they do not share the same ancestor. Thus, our observation of independent origin for the inactivation properties in calcium-selective TRPV channels opens an opportunity to understand in more detail the diversity of mechanisms associated to Ca^2+^ homeostasis in vertebrates.

According to our data, it is likely that the ancestral condition of vertebrates corresponds to a single copy gene. This gene could have had properties similar to the ones observed in contemporary species of cyclostomes, bony fish, and coelacanths, including high selectivity for divalent ions^33^. Gills have a specialized type of cells, called ionocytes, that are responsible for the absorption of ions -including calcium- in both sea and freshwater species^34^. In contrast to terrestrial vertebrates, where kidneys are fundamental for calcium reabsorption, in bony fish and chondrichthyes, this organ is associated with keeping calcium balanced^35^. In bony fish, the decrease of renal calcium reabsorption has been reported as a hallmark for their adaptation to seawater^35^. Our tissue expression data suggest that together with the conquest of land, the repertoire of genes not only expanded but also divided the territories of expression, while one of the copies is mainly expressed in kidneys, others are expressed in a variety of tissues.

Among amniotes, duplicated copies are differentiated by specific amino acid substitutions in both the HLH domain and the S2-S3 linker. These amino acid substitutions are correlated with the phenotype of fast inactivation in all groups of vertebrates but reptiles, where additional modifications -outside of the region analyzed here- might be relevant. The easiness by which the fast inactivation phenotype was introduced make us reason that an additional structural element could even predate the duplication events. Such element might not even be necessarily linked to inactivation when originated. Under this scenario, fast inactivation could be a latent property that readily appeared multiple times in evolutionary history by just introducing specific mutations affecting calcium binding, the correct coupling between putative calcium binding site and the gate, or both. In fact, our data show that calcium-dependent fast inactivation seems to be a general mechanism in vertebrates, present from spotted gar to humans when facing physiological or supra-physiological Ca^2+^ concentrations. Two exceptions where found, anole lizard TRPV6 and chicken TRPV5 channels. Sauropsid physiology requires higher calcium supply when compared to mammals^36^. Viviparity has evolved multiple times in squamates (i.e. snakes and lizards), never in birds, and notably one single time in the common ancestor of mammals^37^. This is thought to be related to the different mechanisms associated to calcium physiology in these species, affecting the evolution of reproduction and early development^36,38,39^. Therefore, would not be unreasonable to suggest that the elimination of calcium-dependent fast inactivation would be beneficial for organisms that require maximizing calcium absorption and reabsorption such as sauropsids. While the elimination of TRPV6 channels in birds is evident according to our data, it would be impossible for us at this point to distinguish between the scenarios originating non-inactivating aTRPV6. Whether the channel evolved to eliminate calcium sensitivity or never evolved to develop such feature would require a larger comparative biology approach that is beyond the focus of the present work.

From a molecular perspective, amino acids at or close to the HLH domain have been implicated in the modulation of temperature-activation in other members of the TRPV family such as TRPV1 and TRPV3^40–42^, and chemical-activation in TRPA1^43^. Molecular dynamic simulations performed on TRPV1 channels suggested that the HLH domain interacts with the intracellular linkers S2-S3 and S4-S5 during gating^44^. In calcium-selective TRPVs, the S2-S3 linker together with residues from the TD-helix and the gate have been associated to the different forms of calcium-dependent inactivation^17,19,22,23^. Therefore, the importance of the structural triad HLH motif - S2-S3 linker - TD-helix in the modulation of channel gating might be extensive to other members of the TRPV family.

## Material and Methods

### Sequence data and phylogenetic analyses

We retrieved calcium-selective TRPV channel sequences from representative species of all major groups of vertebrates. Our sampling included species from mammals, sauropsids, amphibians, coelacanths, holostean fish, teleost fish, chondrichthyes and cyclostomes (Supplementary File 1). Protein sequences were obtained from the Orthologous MAtrix project (OMA)^45^. In cases where the species were not included in the OMA project, we searched the NCBI database (refseq_genomes, htgs, and wgs) using tblastn^46^ with default settings. Protein sequences were aligned using the FFT-NS-1 strategy from MAFFT v.7^47^. We used the proposed model tool of IQ-Tree v1.6.6^48^ to select the best-fitting model of amino acid substitution (JTT + F + R6) (JTT: General matrix)^49^; F: empirical amino acid frequencies from the data; R6: FreeRate model with six categories^50,51^. Phylogenetic relationships were estimated using maximum likelihood (ML) and Bayesian analyses. We employed maximum-likelihood to obtain the best tree using the program IQ-Tree v1.6.6^48^. Support for the nodes was assessed with the Shimodaira-Hasegawa approximate likelihood-ratio test (SH-aLRT), the aBayes test from Anisimova *et al*., 2011^52^ and 1,000 pseudoreplicates of the ultrafast bootstrap procedure^53^. Bayesian analyses were performed in MrBayes version 3.2^54^, running four simultaneous chains for 7 × 10^6^ generations, sampling trees every 2500 generations, and using default priors. We assessed convergence by measuring the standard deviation of the split frequency among parallel runs. Chains were considered to have converged once the average split frequency was lower than 0.01. We discarded trees collected before the chains reached convergence, and we summarized results with a majority-rule consensus of trees collected after convergence was reached. TRPV1, TRPV2, TRPV3, TRPV4 and TRPA1 were used as outgroups.

### Assessment of conserved synteny

We examined genes found upstream and downstream of the TRPV5 and TRPV6 genes in species representative of all major groups of vertebrates. We used estimates of orthology and paralogy derived from the EnsemblCompara database^55^; these estimates were obtained from an automated pipeline that considers both synteny and phylogeny to generate orthology mappings. These predictions were visualized using the program Genomicus v93.01^56^. Our assessments were performed in humans (*Homo sapiens*), chicken (*Gallus gallus*), American alligator (*Alligator mississippiensis*), Chinese softshell turtle (*Pelodiscus sinensis*), anole lizard (*Anolis carolinensis*), African clawed frog (*Xenopus laevis*), coelacanth (*Latimeria chalumnae*), spotted gar (*Lepisosteus oculatus*), zebrafish (*Danio rerio*), and *Callorhinchus milii*, the elephant fish, which is sometimes labeled as elephant shark. In the case of the elephant fish, and American alligator, flanking genes were examined using the Entrez gene database from NCBI^57^.

### Expression analysis

TRPV5 and TRPV6 expression was measured from a representative sample of vertebrates including elephant fish (*Callorhinchus milii)*, spotted gar (*Lepisosteus oculatus*), African clawed frog (*Xenopus laevis*), anole lizard (*Anolis carolinensis*), chicken (*Gallus gallus*) and human (*Homo sapiens*). RNASeq data from a spectrum of tissues including brain, heart, intestine, kidney, liver, muscle, ovary, testis, gills and skin, were gathered from the Short Read Archive (SRA). Accession numbers for species and tissue specific libraries can be found in Supplemental File 1. For all species except the elephant shark and the African clawed frog, we used Ensembl predicted gene sequences for gene expression references. Elephant fish and *X. laevis* cDNA sequences were collected from NCBI. To remove redundancy from libraries, all predicted TRPV5 and TRPV6 transcripts were removed from each library and replaced with our annotations. Adapter sequences were removed from the RNASeq reads using Trimmomatic 0.38^58^, and reads were filtered for quality using the parameters HEADCROP:1, LEADING:30, TRAILING:30, SLIDINGWINDOW:5:30, and MINLEN:50. We used RSEM 1.3.1^59^ to map reads with Bowtie 1.2.2^60^ and to estimate gene expression in units of TPM.

### Molecular biology, cell culture, and transfection

Open Reading Frames (ORF) encoding the different channels analyzed [Spotted gar TRPV5/6 WT (gTRPV5/6), gTRPV5 V294L C300T (SS), anole lizard TRPV5 WT (aTRPV5), aTRPV5 E293D F297L S303T (SS), aTRPV5 I415V F420Y (aTRPV5_VY), anole lizard TRPV6 WT (aTRPV6), aTRPV6 V415M Y420F (aTRPV6_MF), chicken TRPV5 WT (chTRPV5), chTRPV5 E290D F294L S300T (SS), human TRPV5 WT (hTRPV5), hTRPV5 E288D F292L S298T (SS) and human TRPV6 WT (hTRPV6)] were obtained from GenScript Biotech Corporation (Nanjing, China) inserted in a pcDNA3.1(+) vector. HEK 293 T cells were grown in DMEM-F12 medium containing 10% (v/v) bovine fetal serum at 37 °C in a humidity-controlled incubator with 5% (v/v) CO_2_. HEK 293 T cells were transiently co-transfected with the different clones analyzed and peGFP-N1 to allow their identification.

### Electrophysiology and solutions

Whole-cell currents were measured with an Axopatch-200B amplifier. We used borosilicate pipettes (o.d. 1.5 mm, i.d. 0.86 mm, AM-Systems, Squim, WA) with resistance of 2 to 4.0 MΩ to form seals with access resistance between 2 and 6 GΩ. Macroscopic currents were recorded in response to a voltage step protocol from zero to −160 mV. Inactivation was analyzed in a time window of 60 ms. Recordings were digitized at 10 KHz and filtered at 5 KHz using a Digidata 1320 (Molecular Devices, LLC, California). The analysis was performed using Clampfit 10.3 (Molecular Devices, LLC, California). Fast inactivation was assessed by computing the residual currents, defined as the ratio of the current value at the end of the negative pulse over the current at the beginning of the voltage pulse^61^. Inactivation time constants were obtained by fitting the current traces to a single exponential equation. The standard extracellular solution (Ringer-Na^+^) contained (in mM) 140 NaCl, 5 KCl, 2 CaCl_2_, 2 MgCl_2_, 8 glucose and 10 HEPES, at pH 7.4 adjusted with NaOH. Solution for high calcium extracellular concentration (~10 mM free calcium) contained (in mM): 125 NaCl, 10.5 CaCl_2_, 0.5 EGTA and 10 mM HEPES, at pH 7.4 adjusted with NaOH. The standard internal (pipette) solution contained (in mM) 105 CsF, 35 NaCl, 10 EGTA and 10 HEPES, at pH 7.4 adjusted with CsOH. All experiments were performed at room temperature (20 to 25 °C).

### Statistical analysis

Data are expressed as mean ± S.E. Overall statistical significance was determined using analyses of variance (ANOVA one way) with a Bonferroni post-test, and T-student tests. For all conditions, the average was obtained from at least five independent experiments. Outliers were defined by using GraphPad QuickCalcs (https://www.graphpad.com/quickcalcs/Grubbs1.cfm), and removed from the analysis.

## Supplementary information

Supplementary information table 1.

Supplementary information 1.

Supplementary information 2.

Supplementary information 3.

Supplementary information 4.

Supplementary information 5.

Supplementary information 6.

Supplementary figure 1.

Supplementary figure 2.

Supplementary figure 3.

Supplementary figure 4.

Supplementary figure 5.
