# Supplementary figures and images for: Evolutionary analyses reveal independent origins of gene repertoires and structural motifs associated to fast inactivation in calcium-selective TRPV channels
Source: Sci Rep. 2020 May 26;10:8684. doi: 10.1038/s41598-020-65679-6 (PMC7250927; doi:10.1038/s41598-020-65679-6)

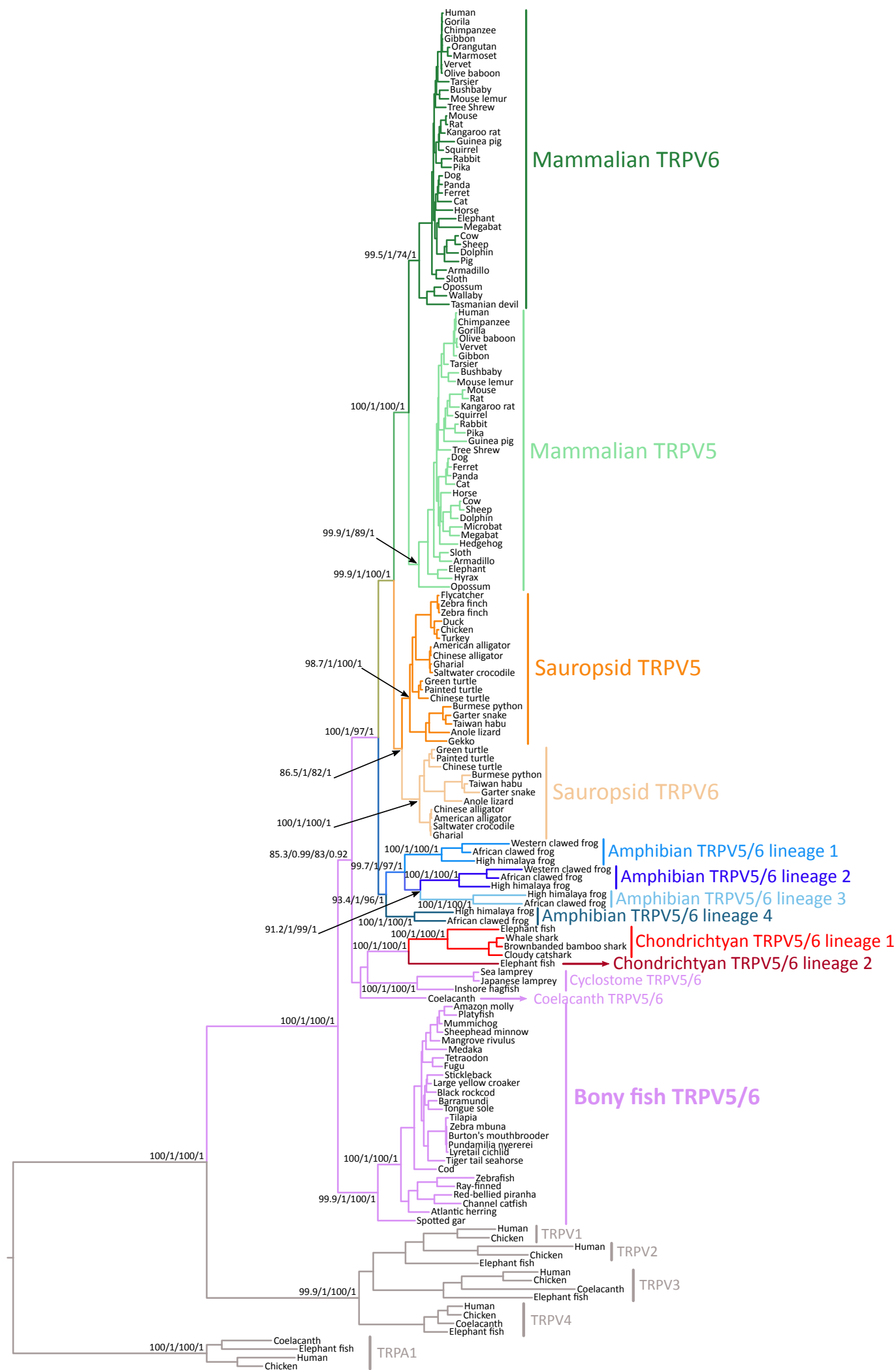

Supplement: Supplementary file 2 — Supplementary information 1. [file 41598_2020_65679_MOESM2_ESM.pdf]

**A**

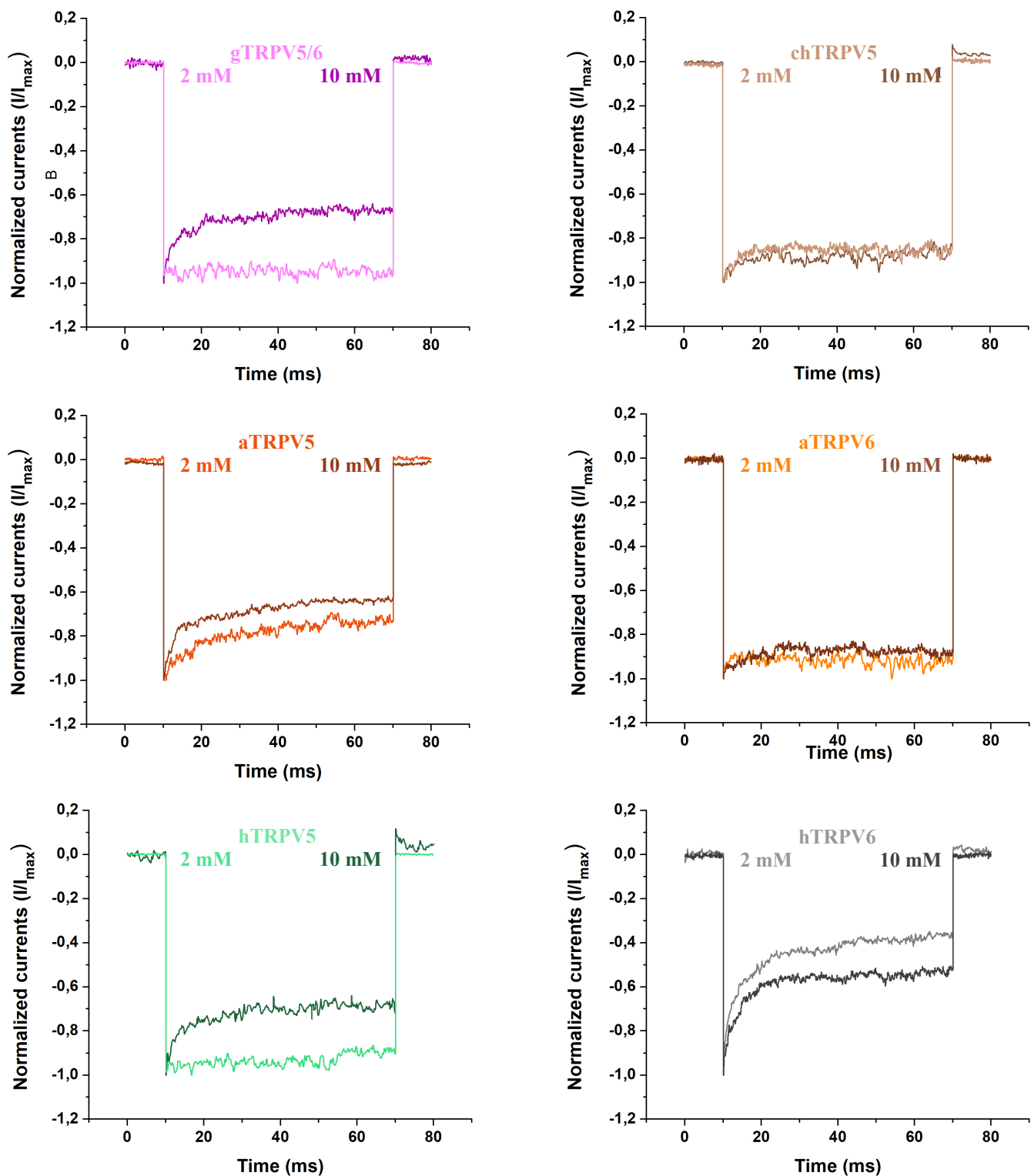

**B**

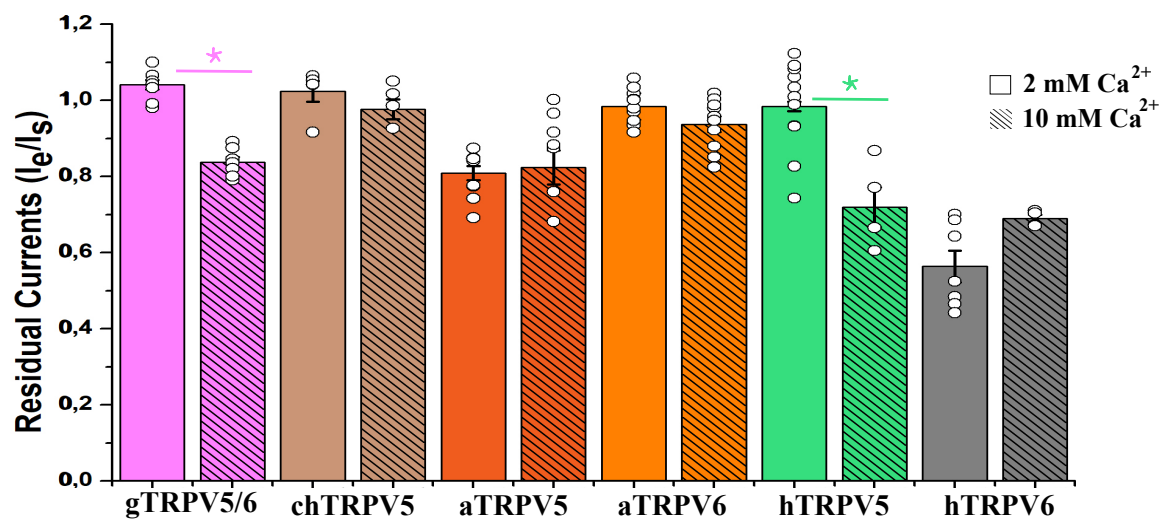

Supplement: Supplementary file 12 — Supplementary figure 5. [file 41598_2020_65679_MOESM12_ESM.pdf]
