# Supplementary material for: Evolutionary analyses reveal independent origins of gene repertoires and structural motifs associated to fast inactivation in calcium-selective TRPV channels
Source: Sci Rep. 2020 May 26;10:8684. doi: 10.1038/s41598-020-65679-6 (PMC7250927; doi:10.1038/s41598-020-65679-6)
Supplement: Supplementary file 8 — Supplementary figure 1. [file 41598_2020_65679_MOESM8_ESM.pdf]

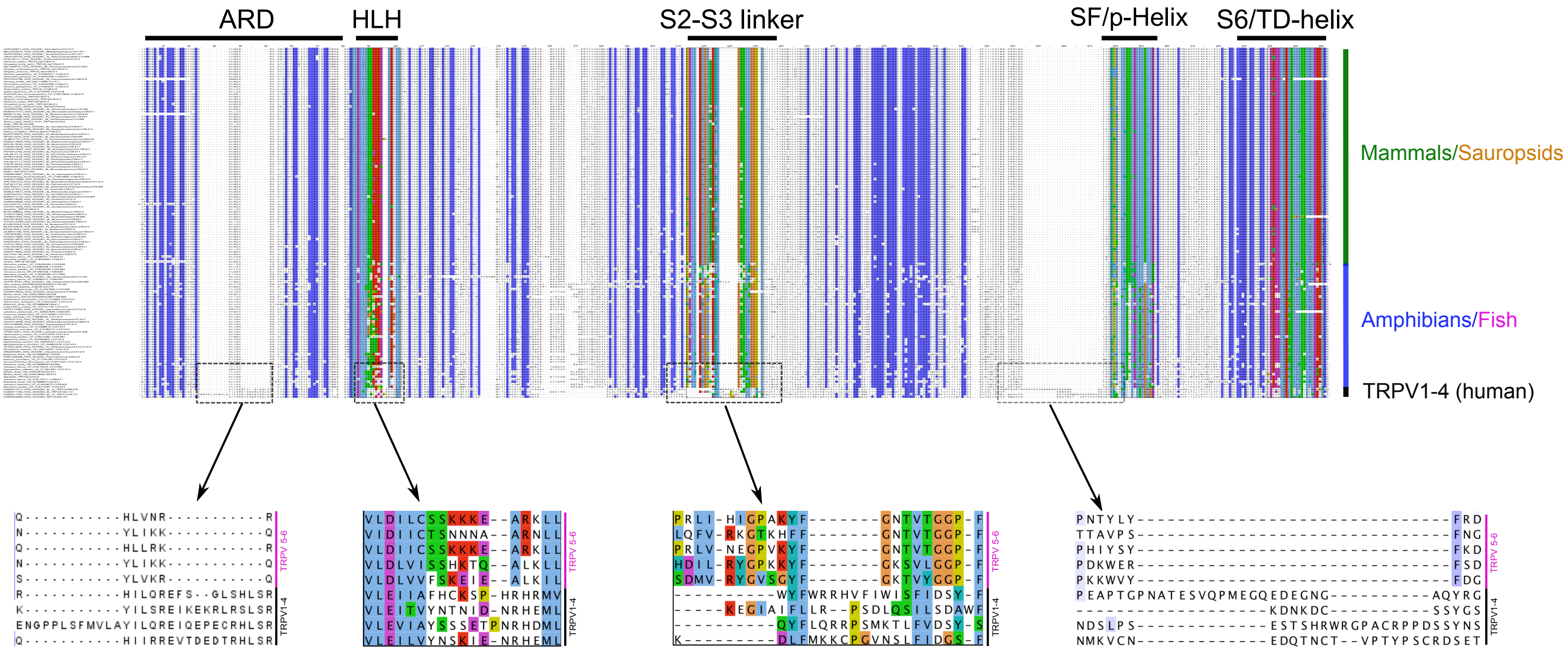

Supplementary figure 1. Amino acid sequence alignment of TRPV5 and TRPV6 channels. Human TRPV1-4 were used as outgroups. Species and genes are denoted to the left and amino acid numbers in the aligment at top. Insets show insertions introduced by TRPV1-4 sequences in the alignment. Relevant channel domains are highlighted on top:

ARD: Ankyrin Repeat Domain

HLH: Helix-Loop-Helix Domain

S2-S3 linker: Intracellular loop between transmembrane segments 2 and 3.

SF/p-Helix: Selectivity Filter & pore Helix

S6/TDhelix: Transmembrane Segment6 & TRP Domain Helix.
