# Supplementary material for: Evolutionary analyses reveal independent origins of gene repertoires and structural motifs associated to fast inactivation in calcium-selective TRPV channels
Source: Sci Rep. 2020 May 26;10:8684. doi: 10.1038/s41598-020-65679-6 (PMC7250927; doi:10.1038/s41598-020-65679-6)
Supplement: Supplementary file 9 — Supplementary figure 2. [file 41598_2020_65679_MOESM9_ESM.pdf]

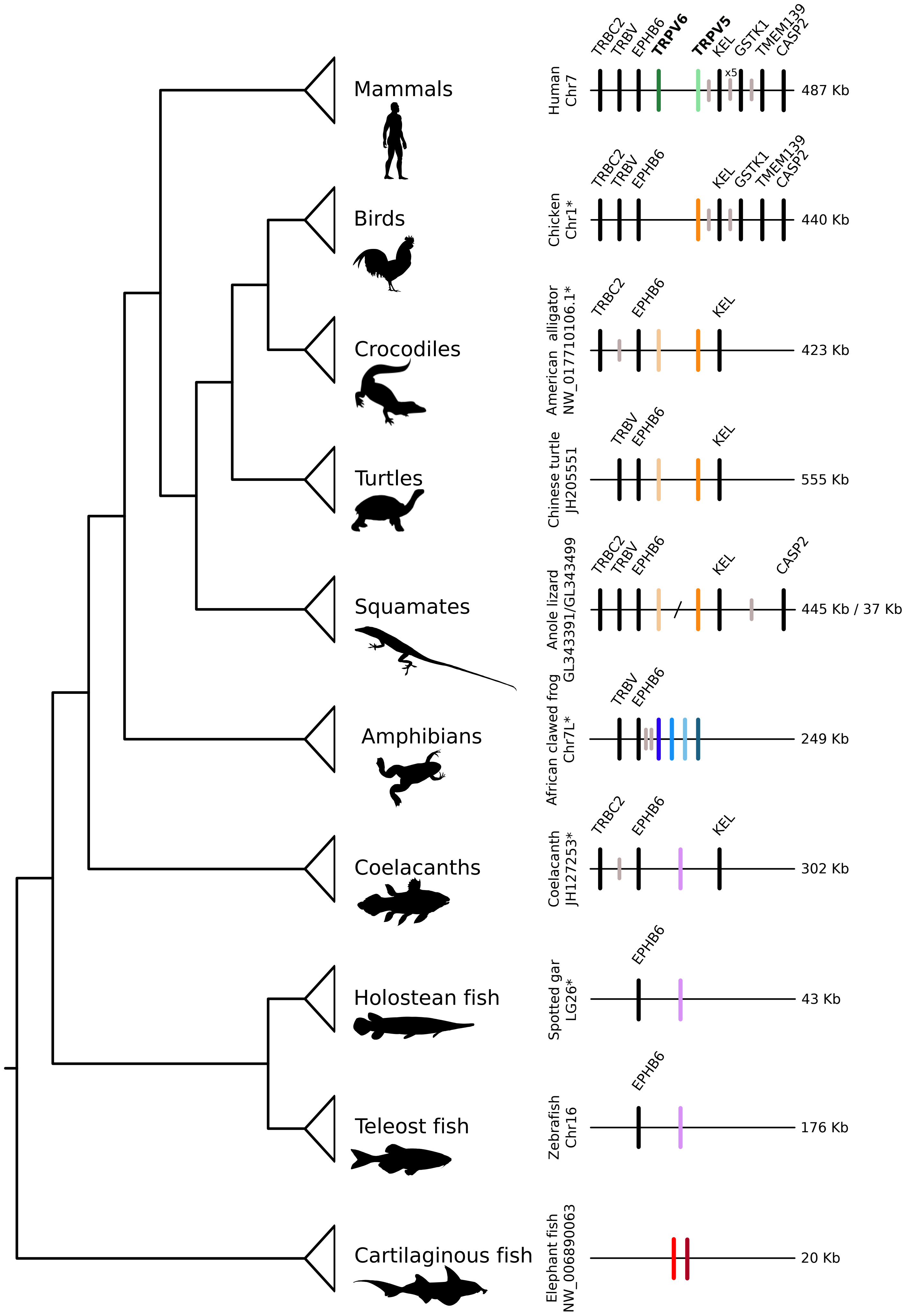

Supplementary figure 2. Patterns of conserved synteny in the chromosomal regions that harbor the calcium-selective TRPV channels of vertebrates. Asteriks indicate that the orientation of the genomic piece is from 3' to 5', gray lines represent genes that do not contribute to conserved synteny, diagonals indicate that the chromosomal pieces are not continuous, whereas numbers on the right denote the size of the chromosomal piece. The colors of the TRPV5 and TRPV6 genes indicate the gene lineage to which they belong according to our phylogenetic tree shown in Fig. 1. Vertebrate silhouette images were obtained from PhyloPic (http://phylopic.org/).
