# Supplementary material for: Evolutionary analyses reveal independent origins of gene repertoires and structural motifs associated to fast inactivation in calcium-selective TRPV channels
Source: Sci Rep. 2020 May 26;10:8684. doi: 10.1038/s41598-020-65679-6 (PMC7250927; doi:10.1038/s41598-020-65679-6)
Supplement: Supplementary file 10 — Supplementary figure 3. [file 41598_2020_65679_MOESM10_ESM.pdf]

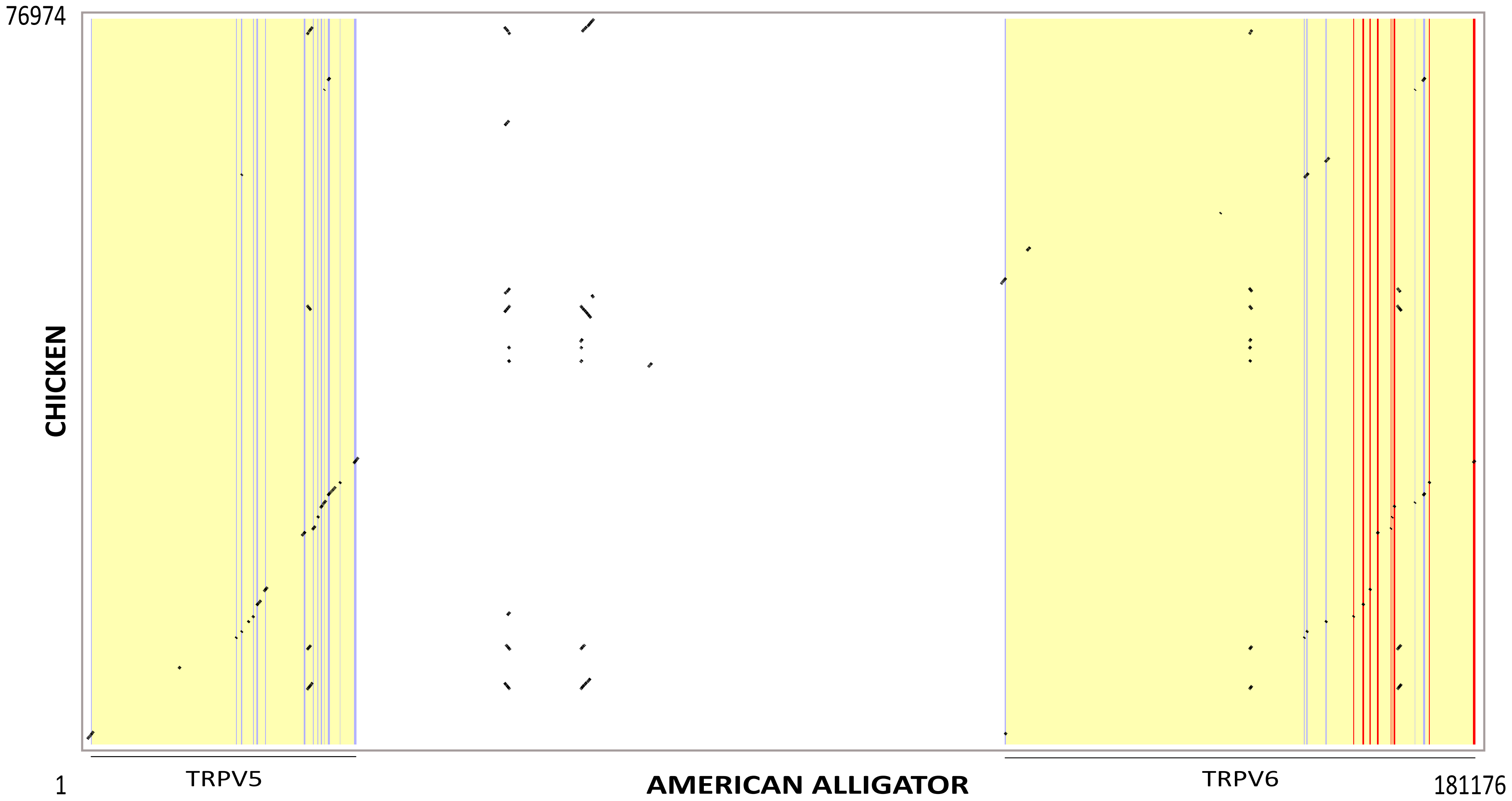

Supplementary figure 3. Dot-plot of pairwise sequence similarity between the TRPV5 and TRPV6 genes of the American alligator (*Alligator mississippiensis*) and the corresponding syntenic region in the chicken (*Gallus gallus*). Light blue and light yellow vertical lines denote exons and introns, respectively. Vertical red lines denote exons that were lost from the chicken TRPV6 gene. Black lines indicate regions of high quality alignment between the American alligator and chicken sequences, and the slope indicates the orientation. All exons of the TRPV5 gene are well conserved, however, for the TRPV6 gene there are several exons (red vertical lines) that do not align between the American alligator and chicken, being consistent with the loss of this gene in birds.
