# Supplementary material for: Evolutionary analyses reveal independent origins of gene repertoires and structural motifs associated to fast inactivation in calcium-selective TRPV channels
Source: Sci Rep. 2020 May 26;10:8684. doi: 10.1038/s41598-020-65679-6 (PMC7250927; doi:10.1038/s41598-020-65679-6)
Supplement: Supplementary file 11 — Supplementary figure 4. [file 41598_2020_65679_MOESM11_ESM.pdf]

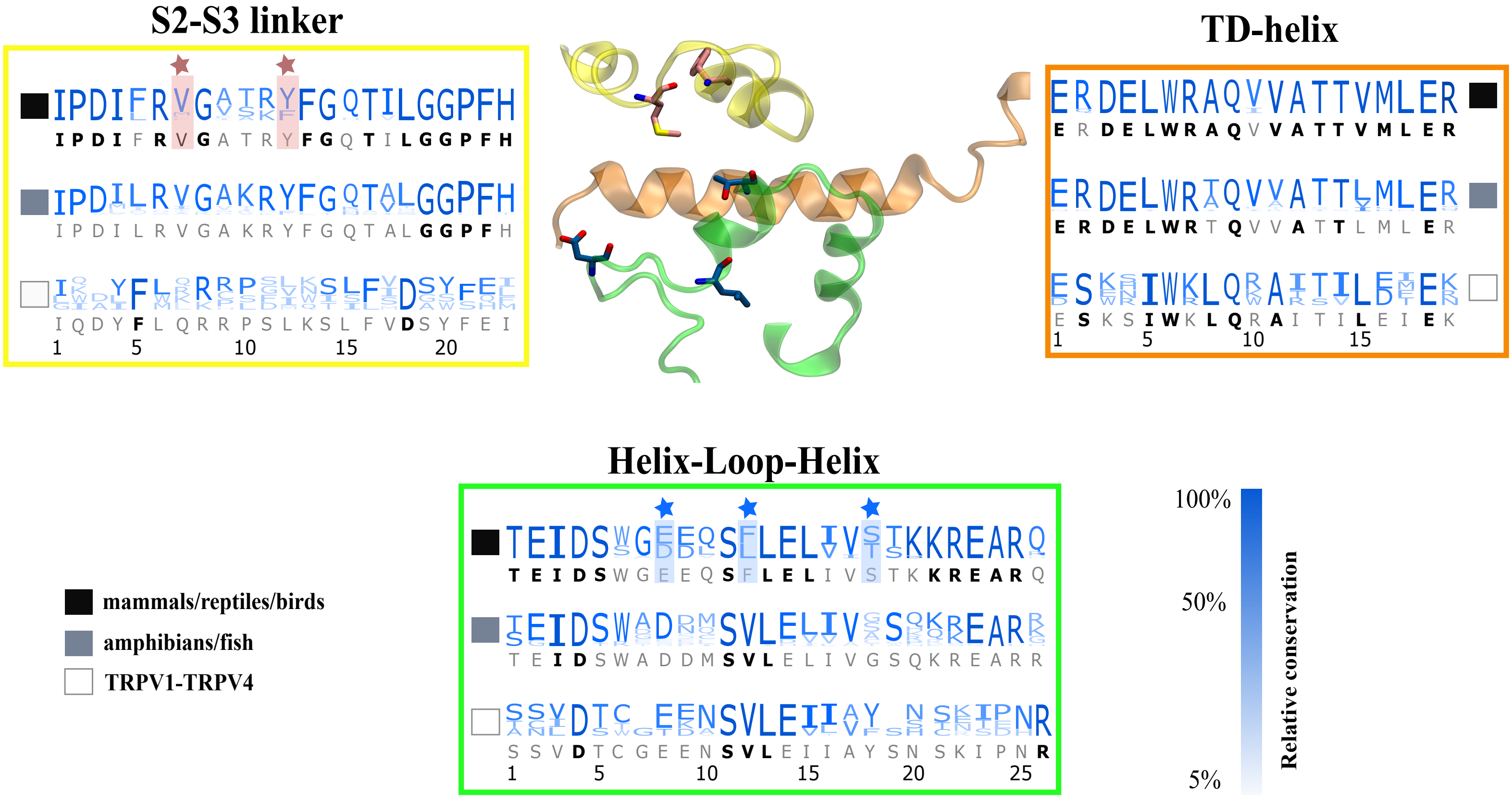

Supplementary Figure 4. Residue conservation (in blue shades) for the components of the structural triad. HLH domain (green), S2-S3 linker (yellow), and TD-Helix (orange) are depicted along with the conservation of three sets: mammalian and sauropsid (black box) data, amphibian and fish (gray box) data and the outer group TRPV1-4 (empty box). Residues highlighted in the S2-S3 linker (light pink shades and asterisk) and the HLH domain (blue shades and asterisk) have been directly related to the inactivation phenotype.
